# Supplementary figures and images for: Dynamics and consequences of the HTLV-1 proviral plus-strand burst
Source: PLoS Pathog. 2022 Nov 28;18(11):e1010774. doi: 10.1371/journal.ppat.1010774 (PMC9731428; doi:10.1371/journal.ppat.1010774)

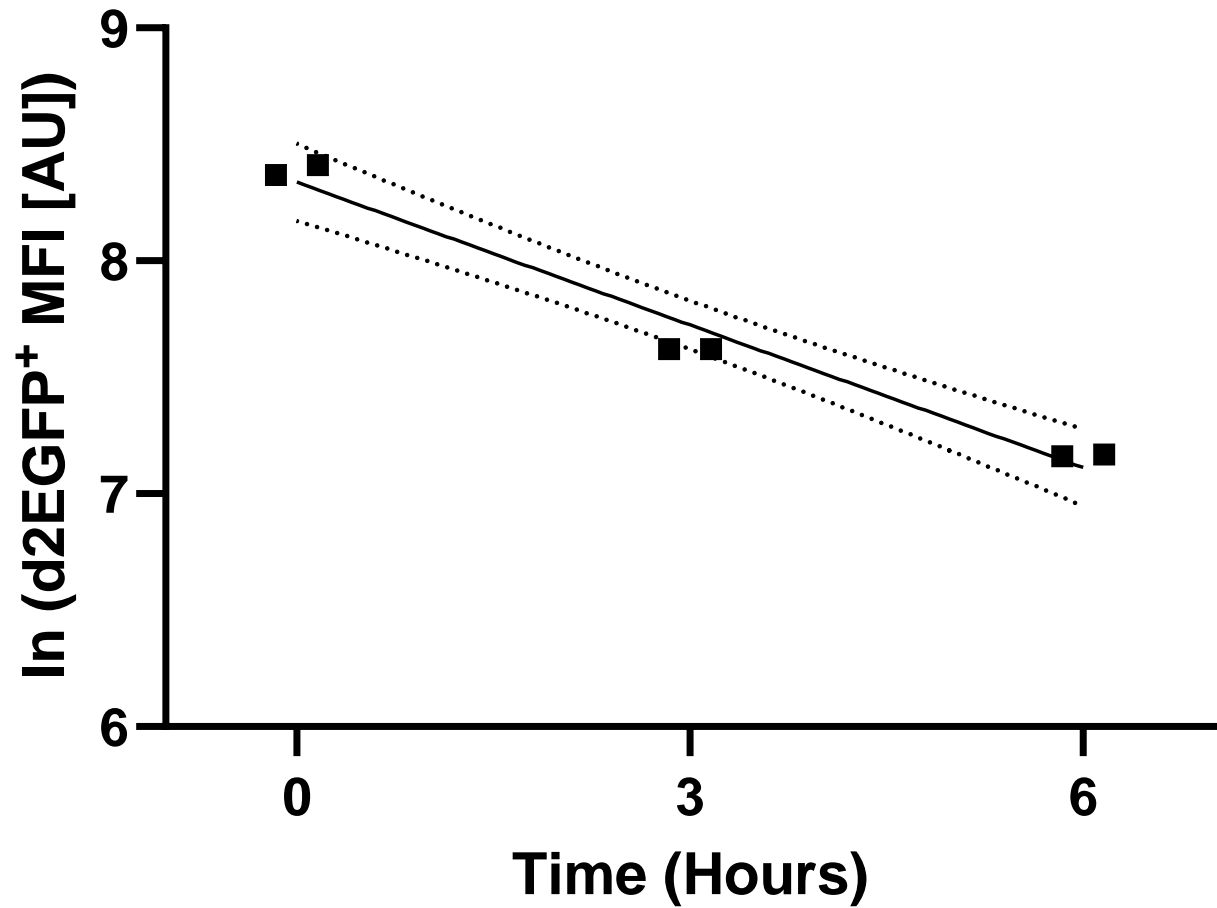

Supplement: S1 Fig — Cells treated with either protein synthesis inhibitor–cycloheximide or vehicle control–DMSO were harvested after 0, 3 and 6 hours of treatment before fixation and flow cytometric acquisition. Natural logarithm-transformed MFI of d2EGFP+ cells was calculated and plotted against time. Technical duplicates from a single experiment using clone d2EGFP TBW 11.50 are shown; the solid and dashed lines show the least-squares regression line and 95% confidence interval, respectively. d2EFP protein half-life was calculated as described in Materials and methods. (PDF) [file ppat.1010774.s001.pdf]

**A**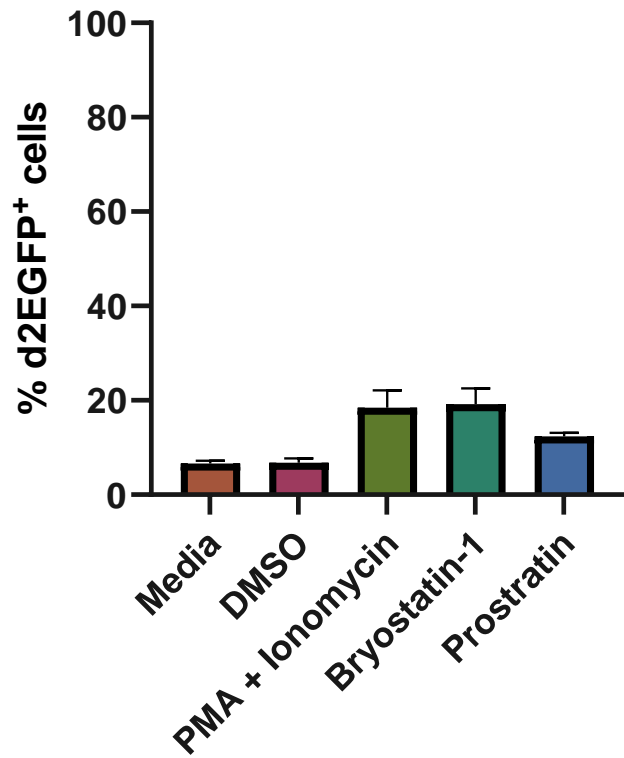**B**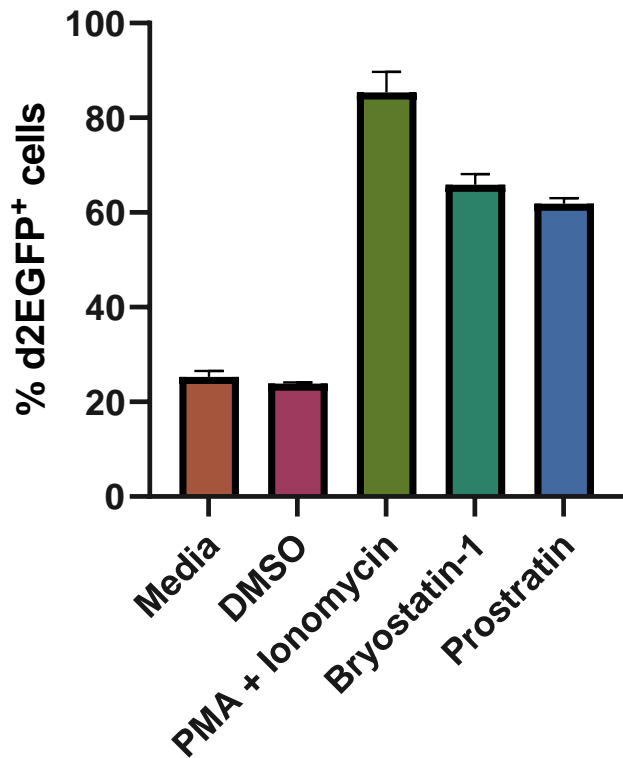

Supplement: S2 Fig — FACS-sorted d2EGFP− cells from (A) d2EGFP TBX4B and (B) d2EGFP TBW 11.50 were cultured with PKC activators bryostatin-1 and prostratin and imaged every 4 hours for 20 hours. PMA and ionomycin were used as the positive control for maximal reactivation, while media alone was used to determine the frequency of cells undergoing spontaneous HTLV-1 plus-strand reactivation. DMSO was used as the vehicle control. The percentage of viable d2EGFP+ (Tax+) cells at the end of the 20-hour culture. Data represent the mean and SEM from two independent experiments. (PDF) [file ppat.1010774.s002.pdf]

**A**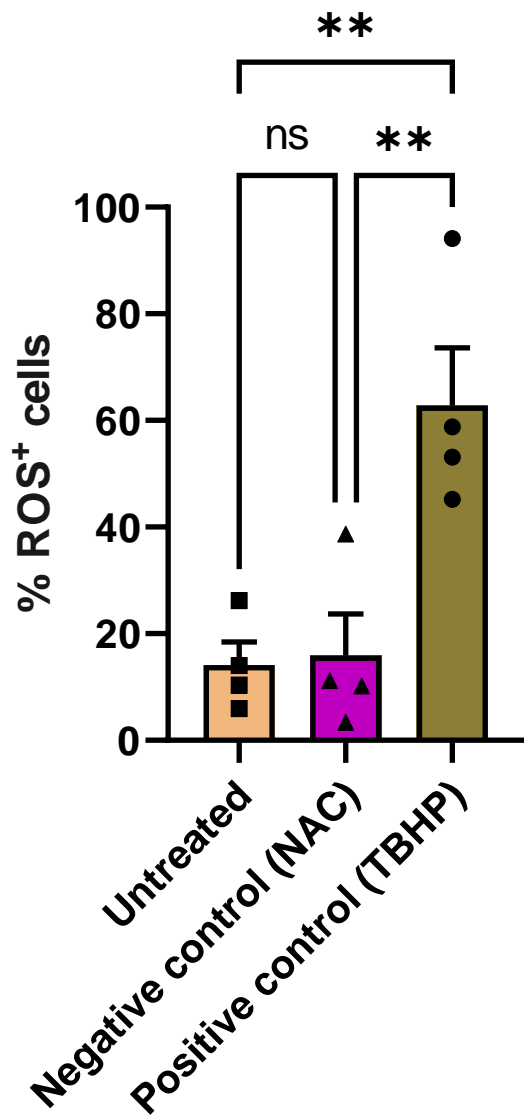**B**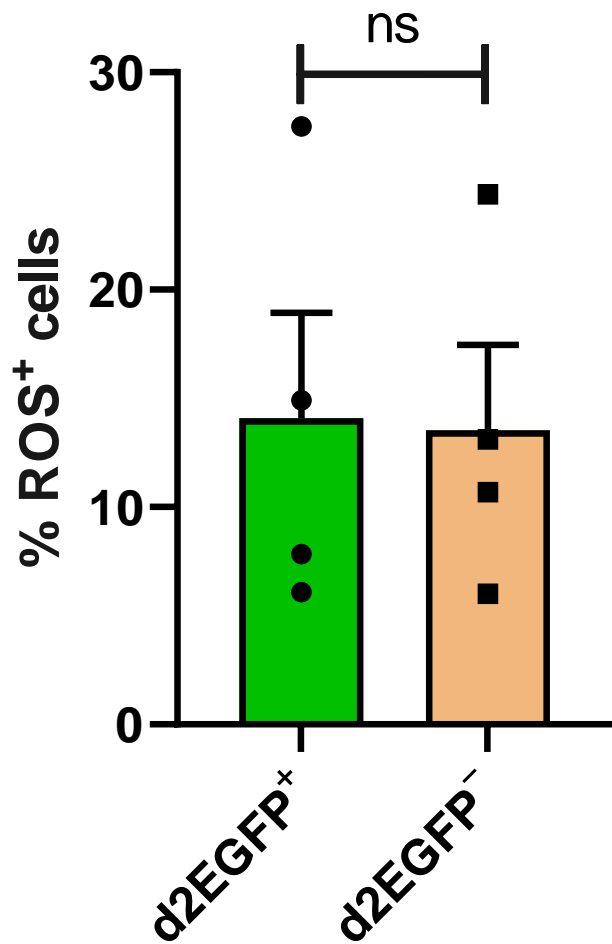

Supplement: S3 Fig — (A) Cells were either untreated or treated with a ROS inducer (THBP) or a ROS scavenger (NAC) for one hour. ROS-expressing cells were detected by flow cytometric analysis after labelling with CellROX Deep Red probe. ROS expression in cells under different treatment conditions. (B) ROS expression in d2EGFP+ and d2EGFP− cells. Data represent the mean and SEM from two independent experiments using the clones d2EGFP TBX4B and d2EGFP TBW 11.50. Statistical analysis of panel A was performed using a one-way analysis of variance (ANOVA) followed by a Tukey’s multiple comparisons test. An unpaired Student’s t-test was used to analyse the data in panel B. ** P < 0.01, ns–not significant. (PDF) [file ppat.1010774.s003.pdf]

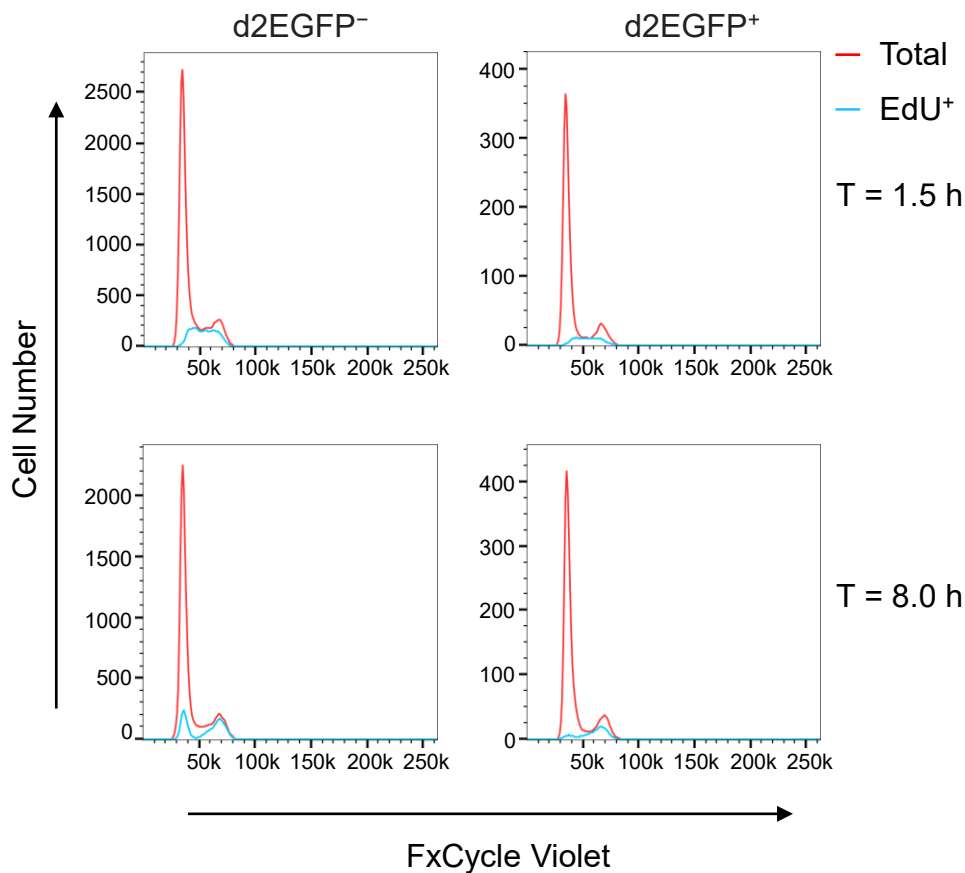

Supplement: S4 Fig — Representative flow cytometric plots of clone d2EGFP TBX4B. (PDF) [file ppat.1010774.s004.pdf]

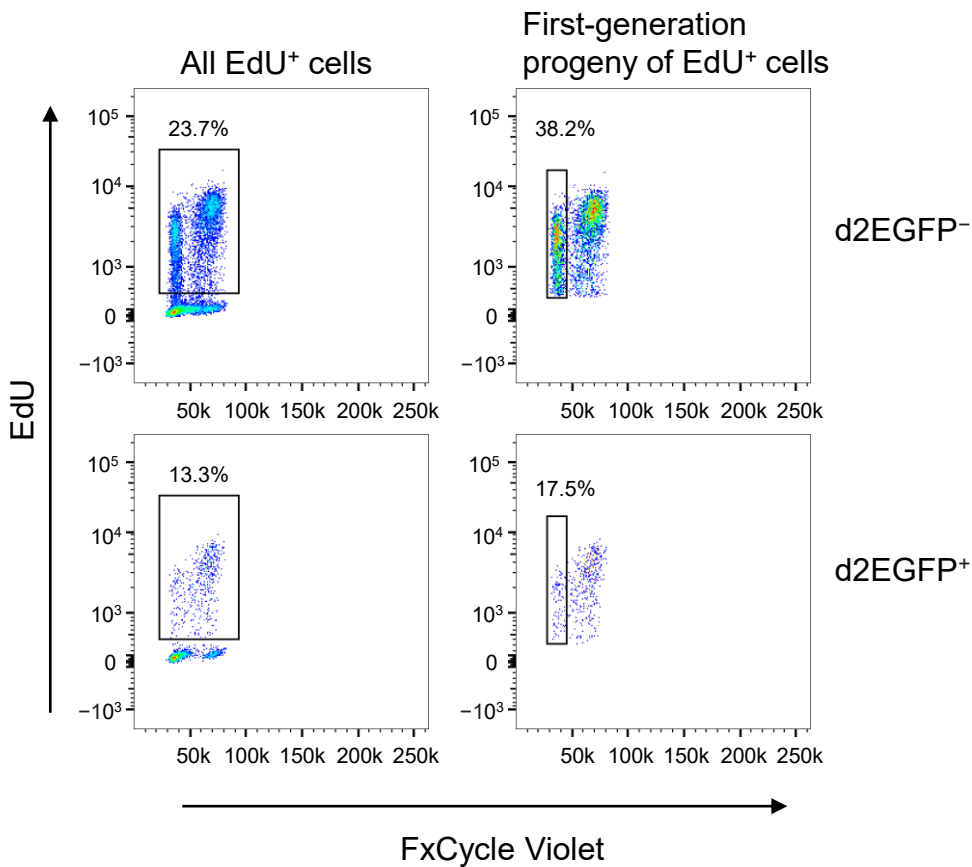

Supplement: S5 Fig — Representative flow cytometric plots of clone d2EGFP TBX4B. (PDF) [file ppat.1010774.s005.pdf]

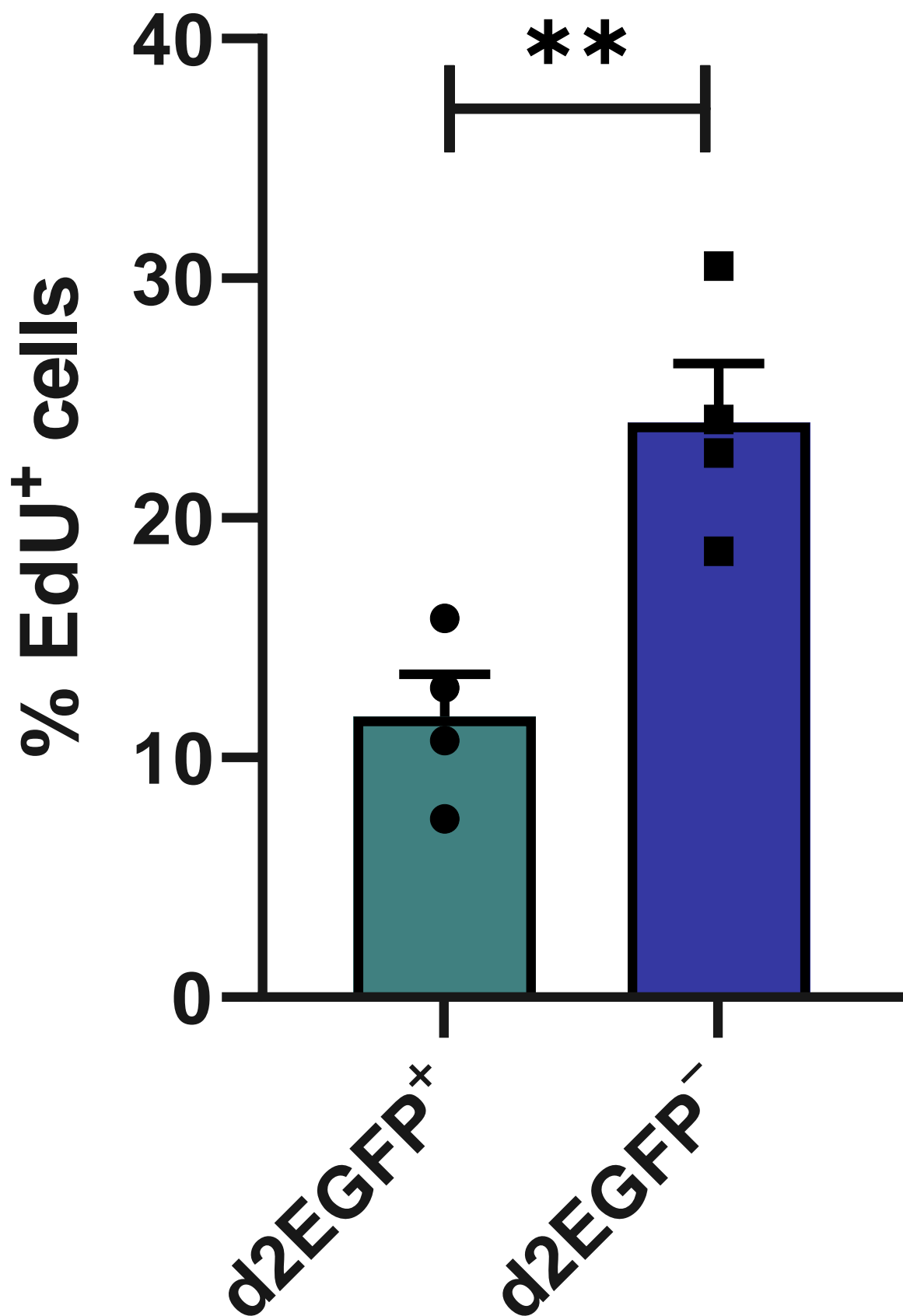

Supplement: S6 Fig — The percentage of cells among d2EGFP+ (Tax+) and d2EGFP− (Tax–) cells that had taken up EdU at the end of the 1.5-hour pulse. Data are mean and SEM from two independent experiments using clones d2EGFP TBX4B and d2EGFP TBW 11.50. Statistical analysis was performed using an unpaired Student’s t-test. ** P < 0.01. (PDF) [file ppat.1010774.s006.pdf]

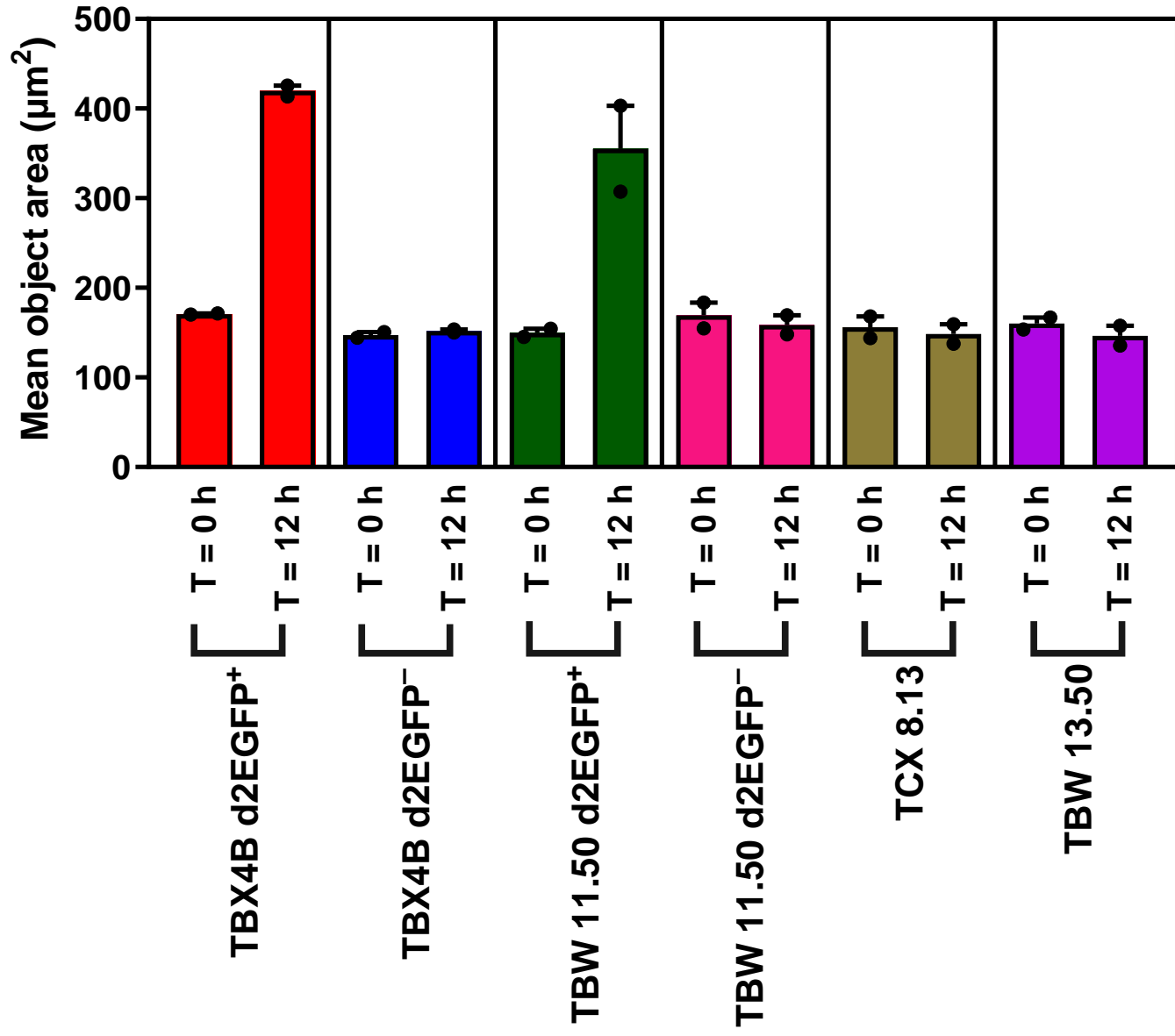

Supplement: S7 Fig — The mean object area of d2EGFP+ (Tax+) and d2EGFP− (Tax–) cells of clones d2EGFP TBX4B and d2EGFP TBW 11.50 and viable cells of clones TCX 8.13 and TBW 13.50 at the beginning (0 h) and end (12 h) of live-cell imaging. The data depict the mean and SEM from two independent experiments. (PDF) [file ppat.1010774.s007.pdf]

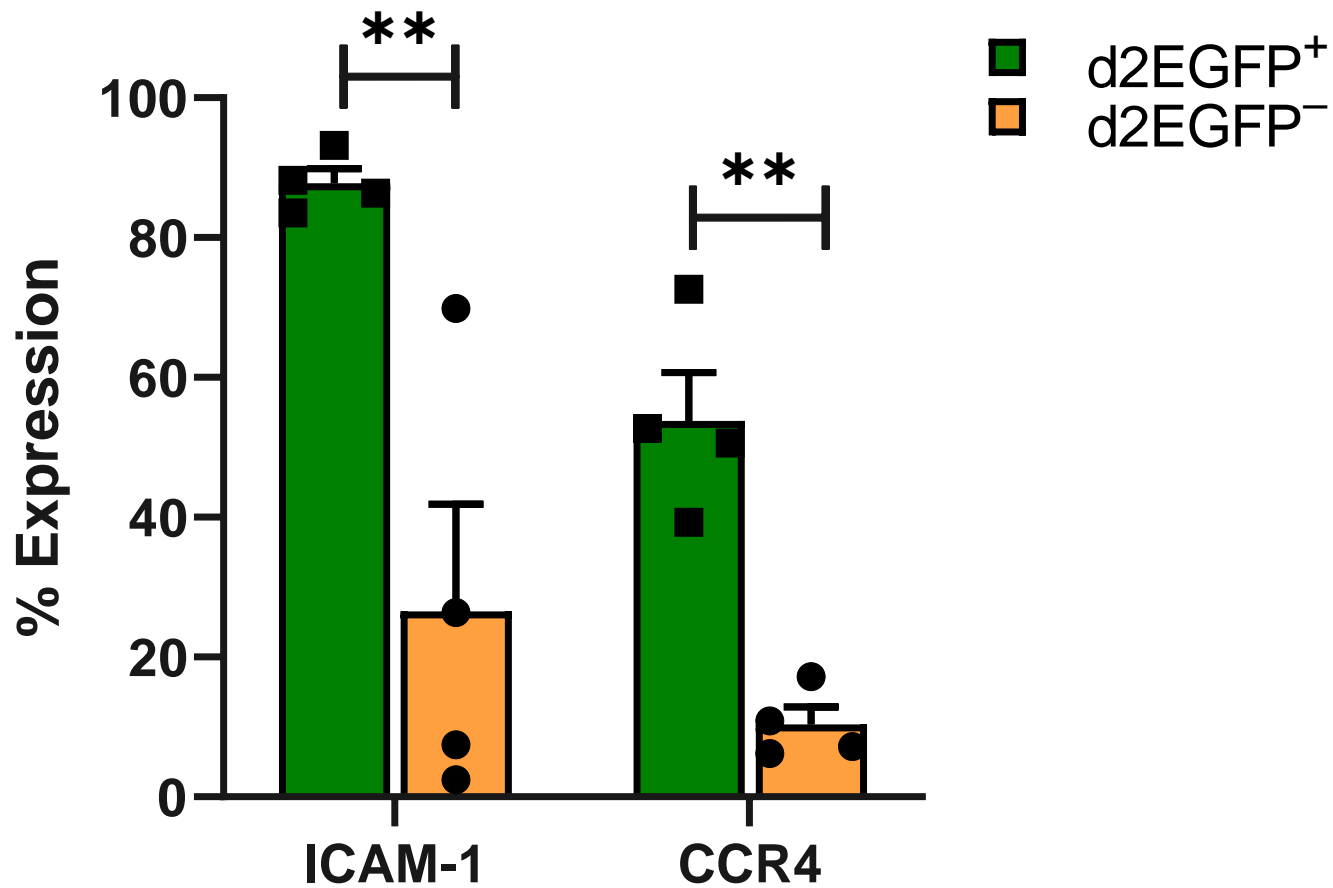

Supplement: S8 Fig — The proportion of d2EGFP+ (Tax+) and d2EGFP− (Tax–) cells that express ICAM-1 or CCR4 was assessed by flow cytometric analysis. Data are mean and SEM from two independent experiments using clones d2EGFP TBX4B and d2EGFP TBW 11.50. An unpaired Student’s t-test was used for statistical analysis. ** P < 0.01. (PDF) [file ppat.1010774.s008.pdf]

**A***tax*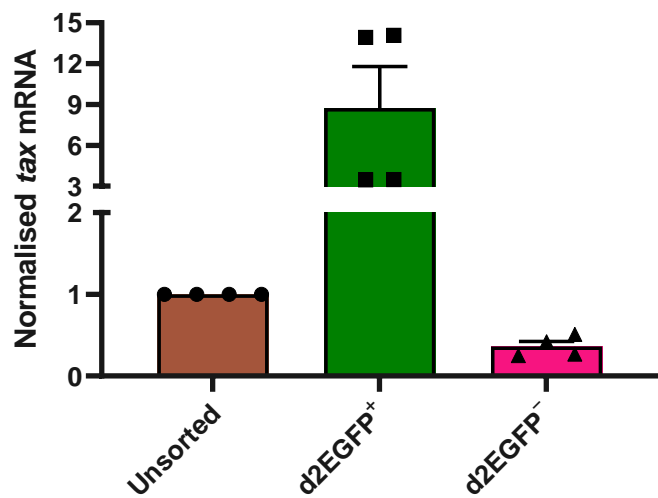**B***d2EGFP*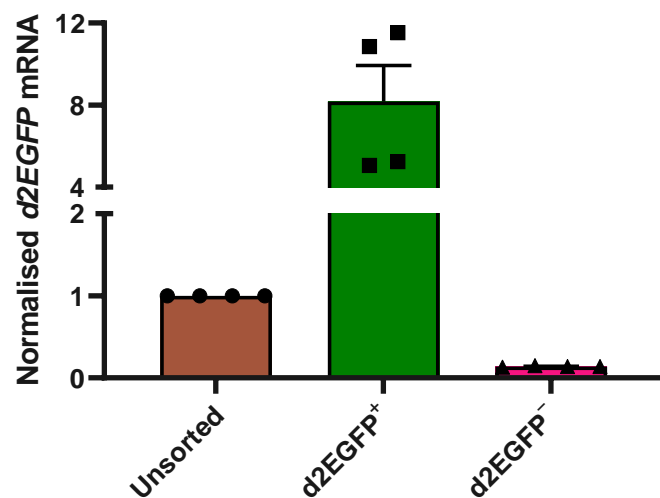**C***CCL22*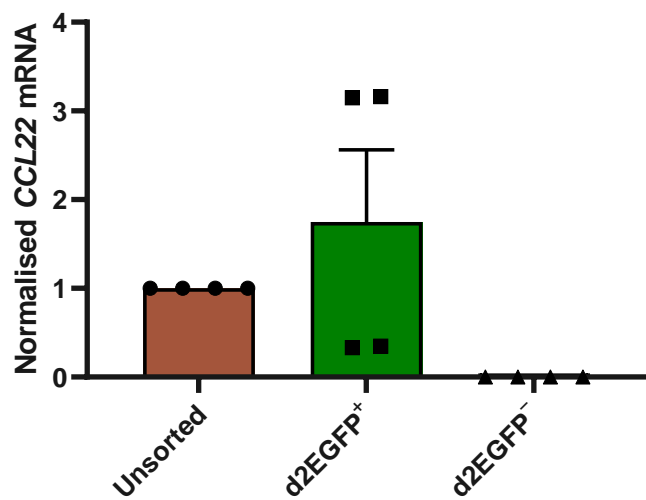**D***sHBZ*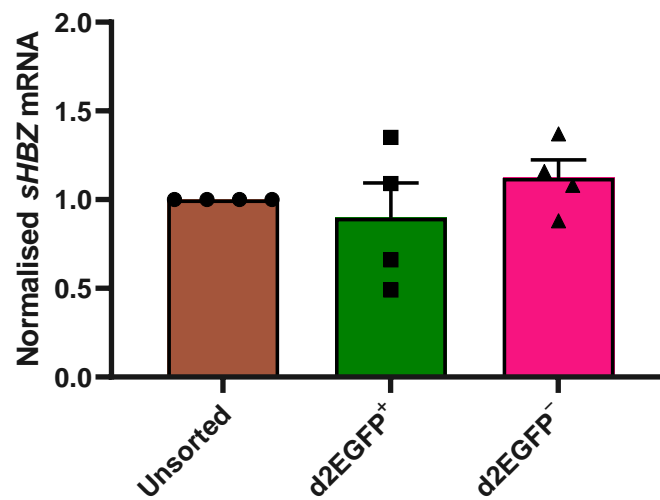

Supplement: S9 Fig — (A) The expression levels of tax, (B) d2EGFP, (C) CCL22, and (D) sHBZ transcripts among unsorted, FACS-sorted d2EGFP+ (Tax+) and d2EGFP− (Tax−) cells were determined using RT-qPCR as described in Materials and methods. The data depict the mean and SEM of two PCR technical replicates of clones d2EGFP TBX4B and d2EGFP TBW 11.50 from a single experiment. (PDF) [file ppat.1010774.s009.pdf]
