# Supplementary material for: Dynamics and consequences of the HTLV-1 proviral plus-strand burst
Source: PLoS Pathog. 2022 Nov 28;18(11):e1010774. doi: 10.1371/journal.ppat.1010774 (PMC9731428; doi:10.1371/journal.ppat.1010774)
Supplement: S1 Table — (DOCX) [file ppat.1010774.s010.docx]

**S1 Table. Details of T-cell clones used in this study.**

| Clone Name | Patient Code | Proviral UIS – GRCh38 | Remarks |
| --- | --- | --- | --- |
| TBX4B | TBX | Chr 22: 43, 927, 318 | Complete single provirus |
| TBW 11.50 | TBW | Chr 19: 27, 791, 679 | Complete single provirus |
| d2EGFP TBX4B | TBX | Chr 22: 43, 927, 318 | TBX4B transduced with PLJM1-LTR-d2EGFP |
| d2EGFP TBW 11.50 | TBW | Chr 19: 27, 791, 679 | TBW 11.50 transduced with PLJM1-LTR-d2EGFP |
| TCX 8.13 | TCX | Chr 16: 53, 567, 147 | Type 2 defective single provirus |
| TBW 13.50 | TBW | NA | Uninfected |

NA – not applicable; UIS – unique integration site
