# Supplementary material for: Dynamics and consequences of the HTLV-1 proviral plus-strand burst
Source: PLoS Pathog. 2022 Nov 28;18(11):e1010774. doi: 10.1371/journal.ppat.1010774 (PMC9731428; doi:10.1371/journal.ppat.1010774)
Supplement: S2 Table — (DOCX) [file ppat.1010774.s011.docx]

**S2 Table. Gene-specific primers used for RT-qPCR.**

| Target gene | Orientation | Sequence | Reference |
| --- | --- | --- | --- |
| *tax* | Forward | 5′-CCGGCGCTGCTCTCATCCCGGT-3′ | [1] |
|  | Reverse | 5′-GGCCGAACATAGTCCCCCAGAG-3′ |  |
| *d2EGFP* | Forward | 5'-AAGCTGACCCTGAAGTTCATCTGC-3' | [2] |
|  | Reverse | 5'-CACCTTGATGCCGTTCTTCTGCTT-3' |  |
| *CCL22* | Forward | 5'-AGGACAGAGCATGGCTCGCCTACAGA-3' | [3] |
|  | Reverse | 5'-TAATGGCAGGGAGGTAGGGCTCCTGA-3' |  |
| *sHBZ* | Forward | 5'-GGACGCAGTTCAGGAGGCAC-3' | [1] |
|  | Reverse | 5'-CCTCCAAGGATAATAGCCCG-3' |  |
| *18S rRNA* | Forward | 5'-GTAACCCGTTGAACCCCATT-3' | [1] |
|  | Reverse | 5'-CCATCCAATCGGTAGTAGCG-3' |  |

Reference:

1. Satou Y, Miyazato P, Ishihara K, Yaguchi H, Melamed A, Miura M, et al. The retrovirus HTLV-1 inserts an ectopic CTCF-binding site into the human genome. Proc Natl Acad Sci U S A. 2016;113: 3054-9. pmid:26929370

2. Martinat C, Bacci JJ, Leete T, Kim J, Vanti WB, Newman AH, et al. Cooperative transcription activation by Nurr1 and Pitx3 induces embryonic stem cell maturation to the midbrain dopamine neuron phenotype. Proc Natl Acad Sci U S A. 2006;103: 2874-9. pmid:16477036

3. Hieshima K, Nagakubo D, Nakayama T, Shirakawa AK, Jin Z, Yoshie O. Tax-inducible production of CC chemokine ligand 22 by human T cell leukemia virus type 1 (HTLV-1)-infected T cells promotes preferential transmission of HTLV-1 to CCR4-expressing CD4+ T cells. J Immunol. 2008;180: 931-9. pmid:18178833
