# Supplementary material for: Dynamics and consequences of the HTLV-1 proviral plus-strand burst
Source: PLoS Pathog. 2022 Nov 28;18(11):e1010774. doi: 10.1371/journal.ppat.1010774 (PMC9731428; doi:10.1371/journal.ppat.1010774)
Supplement: S3 Table — (DOCX) [file ppat.1010774.s012.docx]

**S3 Table.** **Image capturing and analysis parameters used to quantify the frequency of cells undergoing spontaneous and maximal HTLV-1 plus-strand reactivation.**

| Channel | Target cell type | Exposure time (ms) | Method of background fluorescence subtraction | Segmentation parameters |
| --- | --- | --- | --- | --- |
| Phase | All | Not available | Not applicable | Sensitivity (threshold = 9, texture = 10, edge = 10), particle area = 30 – ∞ µm^2^ |
| Green | d2EGFP^+^ | 300 | Top-Hat with 50 µm radius | Not applicable |
| Red | Non-viable | 400 | Top-Hat with 50 µm radius | Not applicable |
