# Supplementary material for: Dynamics and consequences of the HTLV-1 proviral plus-strand burst
Source: PLoS Pathog. 2022 Nov 28;18(11):e1010774. doi: 10.1371/journal.ppat.1010774 (PMC9731428; doi:10.1371/journal.ppat.1010774)
Supplement: S1 Text — (DOCX) [file ppat.1010774.s015.docx]

**S1 Text. Estimation of Tax burst duration using a two-state random telegraph model.**

To estimate the Tax burst duration, we assumed that the burst duration follows an exponentially-distributed random variable with a mean of $1/k_{off}$. This is equivalent to the telegraph process model, where once the burst is initiated, it switches off at the rate $k_{off}$. In this case, if a cell is already undergoing a burst at the start of imaging, the probability of it not switching off after time $T$ is $e^{-k_{off}T}$. For clone d2EGFP TBW 11.50, data across two experimental replicates showed that of the 77 cells that were d2EGFP-positive at the start, 56 remained d2EGFP-positive during 30 hours of live-cell imaging (continuous pattern), while the other 21 exhibited silencing and fluctuating patterns. This corresponds to the probability of continuing a burst to be

$$e^{-k_{off}T}\approx\frac{56}{77}=0.73$$

where $T=30$ hours. Solving this equation yielded the mean Tax burst duration ($1/k_{off}$) to be 94.2 hours. We next used the formula for calculating the confidence interval of a binomial proportion to estimate the lower and upper bounds of this probability. The 95% confidence interval for staying in the d2EGFP-positive state was obtained as (0.63–0.83), which resulted in the 95% confidence interval of the burst duration being (64.4–157.7 hours).

Following a similar analysis for clone d2EGFP TBXB, of the 72 cells that were d2EGFP-positive at the start of imaging, 67 remained d2EGFP-positive during the 30 hours of imaging. This resulted in a mean Tax burst duration of 416.8 hours with a 95% confidence interval of (218.7–2782.0 hours). Note that the few cells turning off led to a much wider confidence interval but still gave a lower bound of 218.7 hours for the burst duration.
