# Supplementary material for: Dynamics and consequences of the HTLV-1 proviral plus-strand burst
Source: PLoS Pathog. 2022 Nov 28;18(11):e1010774. doi: 10.1371/journal.ppat.1010774 (PMC9731428; doi:10.1371/journal.ppat.1010774)
Supplement: Attachment — Submitted filename: 02 Response to reviewers.docx [file ppat.1010774.s017.docx]

There is no file available for this download link.
